# Supplementary material for: Is Fresh Produce in Tigray, Ethiopia a Potential Transmission Vehicle for Cryptosporidium and Giardia?
Source: Foods. 2021 Aug 25;10(9):1979. doi: 10.3390/foods10091979 (PMC8470912; doi:10.3390/foods10091979)
Supplement: Supplementary file 1 [file foods-10-01979-s001.zip › foods-1323336-supplementary.pdf]

Table S1: PCR conditions and primers

Target genes	Primers	Cycling conditions		Reference
*Giardia: s*mall subunit (SSU) rRNA				[1]
First amplification, 292 bp	F: RH11(5′-CATCCGGTCGATCCTGCC-3′)	95°C, 3 min		
	R: RH4 (5′-AGTCGAACCCTGATTCTCCGCCAGG-3′)	96°C, 30 sec<br>59°C, 40 sec<br>72 °C, 40 sec	40x	
		72 °C, 7 min		
Second amplification, 175 bp	F: GiarF (5′-GACGCTCTCCCCAAGGAC-3′)	95°C, 3 min		
	R: GiarR (5′-CTGCGTCACGCTGCTCG-3′)	96°C, 30 sec<br>55°C, 40 sec<br>72 °C, 30 sec	40x	
		72 °C, 7 min		
Giardia: glutamate dehydrogenase (GDH)				[2]
First amplification, 755 bp	F: GDH1 (5′-TTCCGTRTYCAGTACAACTC-3′)	95°C, 3 min		
	R: GDH2 (5′-ACCTCGTTCTGRGTGGCGCA-3′)	94°C, 30 sec<br>50°C, 30 sec<br>72 °C, 60 sec	35x	
		72 °C, 7 min		
Second amplification, 530 bp	F: GDH3 (5′-ATGACYGAGCTYCAGAGGCACGT-3′)	95°C, 3 min		
	R: GDH4 (5′- GTGGCGCARGGCATGATGCA -3′)	94°C, 45 sec<br>54°C, 45 sec<br>72 °C, 45 sec	40x	
		72 °C, 7 min		

***Giardia*: beta giardin (BG)**				[3]
First amplification, 753 bp	F: G7(5′-AAGCCCGACGACCTCACCCGCAGTGC-3′)	95°C, 3 min		
	R: G759 (5′-GAGGCCGCCCTGGATCTTCGAGACGAC-3′)	94°C, 30 sec	35x	
		60°C, 30 sec		
		72 °C, 60 sec		
		72 °C, 10 min		
Second amplification 511 bp	F: (5′- GAACGAGATCGAGGTCCG-3′)	95°C, 3 min		
	R: (5′- CTCGACGAGCTTCGTGTT-3′)	95°C, 30 sec	40x	
		53°C, 30 sec		
		72 °C, 60 sec		
		72 °C, 10 min		
***Cryptosporidium:* small subunit (SSU) rRNA**				[4]
First amplification, 1325 bp	SSU F1: (5′ TTCTAGAGCTAATACATGCG-3′)	95°C, 3 min		
	SSU R1: (5′-CCCATTTCCTTCGAAACAGGA-3′)	94°C, 45 sec	35x	
		55°C, 45 sec		
		72 °C,60 sec		
		72 °C, 7 min		
Second amplification, 840 bp	SSU F2: (5′ GGAAGGGTTGTATTTATTAGATAAAG-3′)	95°C, 3 min		
	SSU R2: (5′-CTCATAAGGTGCTGAAGGAGTA-3′)	94°C, 45 sec	35x	
		55°C, 45 sec		
		72 °C, 60 sec		
		72 °C, 7 min		

References

1. Read, C.; Walters, J.; Robertson, I.D.; Thompson, R.C.A. Correlation between genotype of *Giardia duodenalis* and diarrhoea. *Int. J. Parasitol.* **2002**, *32*, 229–231, doi:10.1016/S0020-7519(01)00340-X.

2. Cacciò, S.M.; Beck, R.; Lalle, M.; Marinculic, A.; Pozio, E. Multilocus genotyping of *Giardia duodenalis* reveals striking differences between assemblages A and B. *Int. J. Parasitol.* **2008**, *38*, 1523–1531, doi:10.1016/j.ijpara.2008.04.008.

3. Lalle, M.; Pozio, E.; Capelli, G.; Bruschi, F.; Crotti, D.; Cacciò, S.M. Genetic heterogeneity at the β-giardin locus among human and animal isolates of Giardia duodenalis and identification of potentially zoonotic subgenotypes. *Int. J. Parasitol.* **2005**, *35*, 207–213, doi:10.1016/j.ijpara.2004.10.022.

4. Jiang, J.; Alderisio, K.A.; Xiao, L. Distribution of *Cryptosporidium* Genotypes in Storm Event Water Samples from Three Watersheds in New York. *Appl. Environ. Microbiol.* **2005**, *71*, 4446–4454, doi:10.1128/AEM.71.8.4446-4454.2005.
